# Introducing RISC: A New Video Inventory for Testing Social Perception

**DOI:** 10.1371/journal.pone.0133902

**Published:** 2015-07-30

**Authors:** Kathrin Rothermich, Marc D. Pell

**Affiliations:** 1 School of Communication Sciences and Disorders, McGill University, Montreal, Canada; 2 Centre for Research on Brain, Language and Music (CRBLM), Montreal, Canada; The University of Nottingham, UNITED KINGDOM

## Abstract

Indirect forms of speech, such as sarcasm, jocularity (joking), and ‘white lies’ told to spare another’s feelings, occur frequently in daily life and are a problem for many clinical populations. During social interactions, information about the literal or nonliteral meaning of a speaker unfolds simultaneously in several communication channels (e.g., linguistic, facial, vocal, and body cues); however, to date many studies have employed uni-modal stimuli, for example focusing only on the visual modality, limiting the generalizability of these results to everyday communication. Much of this research also neglects key factors for interpreting speaker intentions, such as verbal context and the relationship of social partners. *Relational Inference in Social Communication* (RISC) is a newly developed (English-language) database composed of short video vignettes depicting sincere, jocular, sarcastic, and white lie social exchanges between two people. Stimuli carefully manipulated the social relationship between communication partners (e.g., boss/employee, couple) and the availability of contextual cues (e.g. preceding conversations, physical objects) while controlling for major differences in the linguistic content of matched items. Here, we present initial perceptual validation data (N = 31) on a corpus of 920 items. Overall accuracy for identifying speaker intentions was above 80 % correct and our results show that both relationship type and verbal context influence the categorization of literal and nonliteral interactions, underscoring the importance of these factors in research on speaker intentions. We believe that RISC will prove highly constructive as a tool in future research on social cognition, inter-personal communication, and the interpretation of speaker intentions in both healthy adults and clinical populations.

## Introduction

Social dimensions of interpersonal communication have received more and more attention within the last decade, partly due to the rise of social neuroscience [1,2] and the trend to consider pragmatic aspects of language [3]. The meanings encoded by language are often a central feature of social interactions [4,5], although most of our interactions are not entirely literal and require some sort of inference-making ability because what is *said* and what is *understood* are often not the same [6]. Indeed, indirect meanings characterized by irony, sarcasm, and white lies are omnipresent in daily life, although the relationship between the message and speaker intentions is not always the same; for instance, a speaker who is lying means to *hide* their insincerity from the listener, whereas someone employing sarcasm or irony *wants* the listener to recognize the non-literal meaning of their utterance [7]. Thus, the detection of indirect meanings requires careful interpretation of the speaker’s intention, a process relying on the integration of basic semantic and syntactic comprehension, paralinguistic information processing, pragmatic knowledge, visual perspective taking, emotion reading, and theory of mind (ToM). ToM refers to the ability to infer the beliefs, feelings, and intentions of others and is essential for the comprehension of indirect speech and its inference.

The complex nature of nonliteral communication partly stems from its multimodality, since both visual and auditory cues contribute to the perception of indirect meanings; Attardo et al. [8], among others, claim that the recognition of ironic intent is critically reliant on adequate evaluation and integration of cues across different sensory modalities and communication channels. In most instances, the recognition of non-verbal information such as tone of voice, facial expression, and body posture is important to understand indirect meanings that are literally insufficient [9]. Besides these basic cues, external features such as the discourse context have been shown to influence social cognition [10–13], and nonliteral meanings can depend on the type of relationship between communication partners, along such dimensions as closeness/distance and solidarity/authority [14–17].

The ability to recognize indirect meanings and speaker intentions is known to be problematic in a number of clinical populations associated with neurodevelopmental or acquired brain disorder [18–20]. Consequently, there is increasing demand for validated assessment tools with high ecological validity that simultaneously cover multiple facets of social cognition, such as facial expressions, gaze, gestures, body language, and interpretation of contextual clues, which must be processed simultaneously to interpret social behaviors correctly [21–24]. Proper multimodal stimuli that minimally control for semantic content, discourse analysis as well as relationship type are needed to study and assess nonliteral communication under realistic circumstances, but are scarce; other stimuli often omit some of the aforementioned factors that are critical for inferring speaker meaning. One valuable tool for evaluating nonliteral communication was developed by McDonald et al. [25], *The Awareness of Social Inference Test (TASIT*), which evaluates the ability to interpret verbal and non-verbal signals and to judge the mental state of speakers and their specific meaning in conversations. Participants performing the TASIT watch videotaped vignettes, in which actors enact everyday conversations, and then are probed about the emotion of the speaker (Part 1), or their communicative intentions as being sincere, sarcastic or hiding the truth (Parts 2 & 3). Inspired by this work, here we introduce the development of a new database of short videotaped vignettes—the *Relational Inference in Social Communication* (RISC) inventory—which encodes four types of speaker intentions (*sincerity*, *irony (jocularity)*, *sarcasm*, *white lies*) while defining the relationship of communication partners (*romantic couple*, *friends*, *colleagues*, *boss/ employee*) and the availability of discourse context (verbal or physical cues that help to infer speaker meaning). Each vignette represents a verbal exchange between two adults (question-response dyads), some of which begin with a verbal context that reveals the true intentions of the speaker, and participants must interpret the *intended* meaning of the final response. Our main goal was to gather initial perceptual data on these materials through a validation study that would lead to a basic description of the communicative features of the database, and its individual videotaped vignettes, to benefit researchers who may wish to use RISC in studies of social cognition and interpersonal communication. In what follows, we discuss research that informs the various factors that shaped development of RISC and the design of our perceptual validation experiment.

### Speaker intentions

The RISC database incorporates four different types of speaker intentions: sincerity, irony (jocularity), sarcasm, and white lies. Table 1 provides two examples of scripts developed for one two scenarios. *Literal* (or sincere) interactions are marked by a consistency between the meaning of the utterance, the sentiment displayed by the speaker, and the verbal context. Thus, for these items speakers are expressing what they honestly feel or think and are not trying to hide/mask their true opinions or intentions. In order to construct literal and nonliteral utterances with identical lexical content in our database, two distinct literal interactions were formulated; in one case speakers expressed a *positive* disposition towards the topic (*literal positive*) and in the other case they express a *negative* opinion (*literal negative*; see Table 1).

**Table 1:** Examples of scripts corresponding to used scenes in RISC.

Example Scene “Wedding” (with verbal context)	Example Scene “Party” (no verbal context)
*Literal positive*	*Literal positive*
Lisa on the phone: … I’m looking forward already! I’ll call you later!	Anna: Do you think the party was a success?
Paul: Are you gonna come with me to Sarah’s wedding?	Peter: Yeah, I had a great time!
Lisa: Yeah, it is gonna be fun!	
*Literal negative*	*Literal negative*
Lisa on the phone: … and I don’t really wanna go there, anyways. I’ll call you later!	Anna: Do you think the party was a success?
Paul: Are you gonna come with me to Sarah’s wedding?	Peter: No, no one fun.
Lisa: No, weddings aren’t really my thing.	
*Sarcasm*	*Sarcasm*
Lisa on the phone: … and I don’t really wanna go there, anyways. I’ll call you later!	Anna: Do you think the party was a success?
Paul: Are you gonna come with me to Sarah’s wedding?	Peter (sarcastic): Yeah, I had a great time!
Lisa (sarcastic): Yeah, it is gonna be fun!	
*Jocularity*	*Jocularity*
Lisa on the phone: … I’m looking forward already! I’ll call you later!	Anna: Do you think the party was a success?
Paul: Are you gonna come with me to Sarah’s wedding?	Peter (jocular): No, no one had fun.
Lisa (jocular): No, weddings aren’t really my thing.	
*White lies*	*White lies*
Lisa on the phone: … and I don’t really wanna go there, anyways. I’ll call you later!	Anna: Do you think the party was a success?
Paul: Are you gonna come with me to Sarah’s wedding?	Peter: Yeah, I had a great time!
Lisa: Yeah, it is gonna be fun!	

Compared to sincerity, *irony* is an indirect form of speech characterized by an opposition between the literal meaning of an utterance and the intended meaning [26]. We decided to focus on one particular form of irony, hereafter referred to as “jocularity”, which is often described as “positive” sarcasm, banter, mocking or teasing [16,27]. In the current database, jocular comments are *negative* statements with *positive* intentions. These statements are considered risky because they can be taken as direct insults; for instance, if you say “You’re a terrible cook!” to your communication partner, he/she could miss your ironic intent and be seriously offended as a result.

Thus, it is crucial for the speaker use paralinguistic cues to be sure such “gentle mockery” is perceived as such [28]. Research suggests that the nature of the relationship (close versus distant) might be particularly important for understanding jocular utterances [29]. When successful, it is believed that jocularity allows individuals to enhance their bonds through the indirect expression of affection and shared laughter; this seems to be especially true if the individuals are close enough to tease without harming the relationship [27,30]. Indeed, the most common cues to signal jocularity are laughter and linguistic phrases, such as "*Just kidding*!“, to ensure that the listener does not interpret the comment in the wrong way [27]. Smoski and Bachorowski [31] observed that laughter cues in the context of jocularity seem to have a dual function: to mark the presence and understanding of jocularity, and to reinforce social relationships.

*Sarcasm* has been described as a form of verbal irony that is bitter and directed *against* an individual [28,32], and in opposition to jocularity it refers to *positive* statements with *negative* intentions (for example saying “You are a great cook!” to somebody who messed up a dinner meal). One of its possible social functions is to change someone’s behavior or opinion through a polite form of criticism [33] or to increase the perceived politeness of the speaker [34] and to mitigate the aggressiveness of the critical comment [35]. However, other studies imply that the social function of sarcasm is oftentimes to be humorous [36] or to heighten dramatic effect [37]. In terms of cues, it is difficult to define sarcastic and ironic cues in separation, since these terms are often used interchangeably in the literature; Attardo et al. [8] described a number of visual markers of jocularity/sarcasm when examining American actors in sitcoms on television. These include: raised or lowered eyebrows; open, squinting or rolling eyes; winking, nodding or smiling; or no expression at all (i.e., blank face). For example, Williams et al. [38] found that speakers divert their gaze when being jocular/sarcastic in conversations. Besides visual cues, vocal cues to sarcasm include slowed speech rate and nasalization [39], restricted pitch variation and long pauses between words [40], and, most consistently, changes in pitch-contour [40–44]. Differences in pitch are frequently identified as a major marker of sarcasm; however, the manner in which pitch appears to be used (i.e. higher, lower or monotonous) varies between studies [43], Attardo, et al. [8] argue that there is no single ironic tone of voice and that vocal cues are simply based on a departure from an individual's normal speech pattern.

*Lies*, the act of intentionally trying to mislead another person, are also a frequent part of everyday life [45]. Although lies can be told for personal gain, the majority seem to arise for psychological reasons related to “saving face”: to avoid hurting another’s feelings; to be accommodating and make things easier or more pleasant for others; to protect others from loss of status or position; or to protect others from bother/doing something they prefer not to do [45–48]. We refer to these as *white lies* when they function to modulate one’s self-image and others’ perception of behavior. White lies, which involve hiding one’s true feelings or evaluations, are commonly told to ensure that a social interaction proceeds smoothly [49–51] and to avoid negative consequences for the target [46]. Other social reasons include a desire to avoid tension or conflict, to preserve interpersonal relationships, and to achieve interpersonal power [46,52].

In comparison to jocularity and sarcasm, white lies seem to be triggered by a complex set of motivations and intentions that render them expected in certain social situations. Thus, *not* producing a white lie when expected can violate social norms and produce tension between communication partners, even more than jocularity/sarcasm in many situations. While speakers usually try to hide the fact that they are lying, various auditory and visual cues that accompany the production of white lies can “leak out”; for example, auditory cues include temporal fluctuations and increased pitch [53,54]. Recently, Rigoulot et al. [55] found that white lie utterances are higher in pitch and demonstrate more pitch variation than corresponding sincere utterances. In addition, white lies may be accompanied by diverting eye contact [56] or gaze avoidance, increased blinking, postural shifts, speech errors, and speech hesitations [57–59]. By including social exchanges in our inventory that lead to a white lie, we expect that many of these cues will be captured in our stimuli and used to differentiate white lies from other literal and nonliteral speaker intentions.

### Social relationships

Another important aspect of social interactions is the *type* of relationship between communication partners, for example intimate or business. This factor plays an essential role in whether literal and nonliteral comments are expected and how they are perceived [16]. Imagine a boss and her employee talking about a recent conference, and the boss asking if she liked it. Let us assume the employee did not like the conference at all; she now has several options how to answer this question. She could be literal, but in the event that her boss was somehow involved in organizing the conference (or in another similar scenario), responding in an honest (literal) manner might not be well received. Instead, the employee is likely to utter a white lie and pretend that she liked the conference in these circumstances. However, in a similar situational context with a more familiar communication partner (e.g., a friendly colleague), the employee might answer the same question ironically, with the intention of being humorous, or sarcastically to mark criticism. This exemplifies that what is expected and tolerated in a communicative setting depends highly on familiarity and whether the relationship is defined as close, casual, solidary (i.e., individuals who share the same interests) or authoritarian [17].

In their overview of how relationships shape jocularity and sarcasm perception, Pexman and Zvaigzne [29] emphasize that close, liking relationships are more prone to use sarcasm than other types of relationships. This conclusion fits with Gibbs’ [27] observation that friendship seems to encourage ironic talk, which may improve close relationships by emphasizing the shared background of the participants [16,34]. Kreuz [60] underscored a link between the use of jocularity and relationship type, proposing the term *inferability* to signify that people are more likely to use jocularity in situations in which they are sure it will be understood as intended; when speaker and listener know each other well, they are better able to foresee how a statement will be perceived. As Clark and Gerrig [61] point out, jocularity can be accomplished only when speaker and listener share relevant knowledge (or “common ground”); thus, jocularity is used more often and understood more readily in close relationships. Indeed, Kreuz [60] found a positive correlation between ratings of closeness and ratings of the likelihood of using sarcasm with target individuals, suggesting that people are more likely to use sarcasm in close relationships than in distant relationships [34].

It has also been shown that lying depends on the type of relationship, the type of lie, as well as an interaction between the two. In general, lies become more socially problematic in close when compared to casual relationships [47,62]. In non-intimate relationships, Hample [63] concluded that lies are told to more powerful others (employers, parents, teachers) in order to defend oneself, while Camden et al. [46] found that subjects lied to satisfy their basic needs, manage affiliation, and protect their self-esteem. More frequently, *white lies* are told to close family members [47] and other people who are emotionally invested in the content of the lie [64]. There is contradictory evidence as to how much white lies are expected or tolerated in different relationships. On one hand, an individual intent on being totally honest in close relationships could face negative consequences due to increased social conflict [65]; however, on the other hand, research by Levine and Schweitzer [48] reveals that individuals who tell altruistic (white) lies are perceived as more moral than those who tell selfish truths. Certainly, lying behavior threatens interpersonal trust [66] and can impede the resolution of intimate conflict [67]. However, it should be kept in mind that in certain social-relational contexts, *literal* interactions can also violate social norms [68,69].

### Context

It is well known that both verbal and nonverbal information play an essential role in the creation and comprehension of the communicative message as a whole [36,70]. Nonliteral language, in particular ironic utterances, is often used to explore the effects of a supportive context on deriving contextually suitable interpretations [71], for example by manipulating context strength in various ways. Of note, Katz and colleagues [72–74] introduced discourse elements that evoked expectations of either a literal or a nonliteral interpretation as a function of the speaker’s occupation; their results suggest that with appropriate contextual support, the nonliteral (sarcastic) meaning of a statement is made available as rapidly as the literal meaning. This claim is in line with results showing that as discrepancies between an utterance and its factual context become more obvious/pronounced, listeners are more likely to arrive at a nonliteral interpretation of the utterance [75].

Thus, there are strong indications that the categorization of speaker intentions is guided by contextual knowledge, such as verbal and physical cues, that reveal the speakers’ true state of mind [25]. Accordingly, in the present study, verbal and physical context was systematically introduced to furnish information about the true opinions and feelings of the speaker, and to a lesser extent as a cue to *specific* speaker intentions. In some cases (e.g., literal versus sarcastic vignettes), the verbal context that precedes the literal or nonliteral statement is identical and should therefore not bias participants’ interpretation of the final utterance; in other settings, contextual cues (e.g., literal versus white lies) play an important role in understanding the speaker’s cognitive state and for recognizing speaker intentions, a factor that will be monitored in our results.

### Current approach

Based on our discussion, development of the RISC database was meant to be sensitive to different relationship manipulations marking social distance (couple, friends, colleagues, boss/employee) that were played by four actors who communicated different sets of intentions (literal, jocular, sarcastic, white lie). Although we controlled the verbal script that actors could use to communicate different intention types, our goal was to elicit relatively naturalistic interactions among the participants where intended meanings were expressed using a range of vocal, facial, gestural and whole body movements that felt appropriate for the situational context. Our goal was to gather information about how well speaker intentions were recognized by a group of participants who categorized each video in a forced choice response paradigm; these data could then be used to generate a comprehensive, controlled set of recordings that encode different speaker intentions for future empirical work. Our general expectation was that vignettes encoding literal meanings would be identified more accurately than nonliteral meanings [72,76,77], since nonliteral meanings and white lies rely in a more complex manner on concomitant social and contextual cues (e.g., integration of nonverbal cues with the verbal message, integration of preceding verbal information that conveys the speaker’s true opinion). We further anticipated that sarcasm and jocularity would be recognized more effectively in close relationships (couple, friends) than in distant relationships (boss-employee), yielding significant differences in accuracy according to relationship type to recognize these (and possibly other) intentions.

## Methods

### Production study

#### Ethics Statement

This study was ethically approved by the McGill Faculty of Medicine Institutional Review Board in accordance with principles expressed in the Declaration of Helsinki. Informed written consent was obtained from each participant prior to their involvement in the research, and additional consent to publication was obtained prior to publication.

#### Participants

Four native English speakers (two male, two female; mean age in years: 19.50, SD: 0.50), recruited for having amateur acting experience (mean years experience: 4.00, SD: 0), expressed the different communicative intentions. None of the actors were acquainted prior to taking part in the study and each received CAD $10 per hour as compensation. All actors spoke with a standard (central) Canadian English dialect.

#### Materials

**Fig 1:**
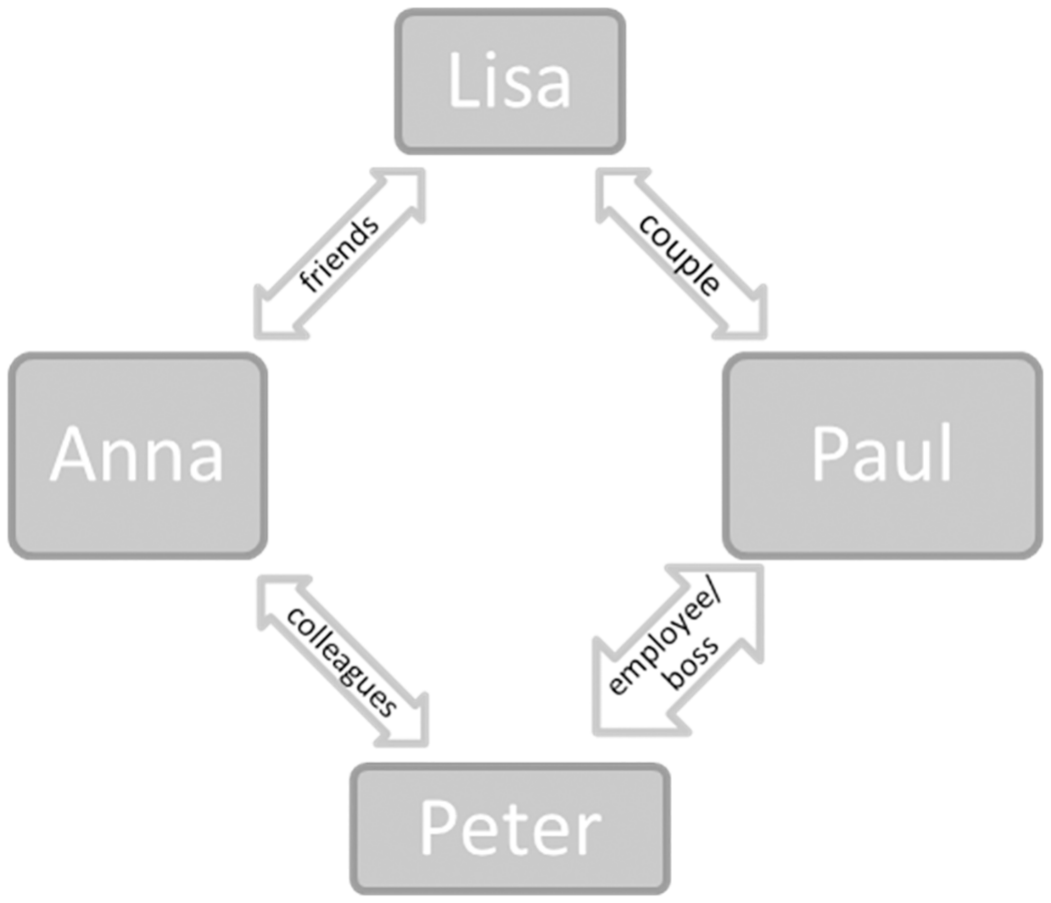
Character relationships. Structure of the relationship among the four characters who communicated different intentions in the RISC vignettes.

Forty-eight different scenes, or social-interactive contexts, were constructed in which actors communicated four different speaker intentions (literal, jocularity, sarcasm, white lie) in each of four relationship types (defined below). As a basic structure, each script included an invariable question posed by one of the two characters (e.g., “Did you like that restaurant I recommended?”), which was followed by one of two possible responses from the other character to allow each of the four intentions to be communicated in the context of the scene. One of the two responses was positive (“The food was exceptional!”) and one was negative (“It was a bit over the top for me”). Positive responses could be expressed in a literal positive manner, sarcastically or as a white lie. Negative responses could be delivered in a literal negative or jocular manner.Each of the four actors assumed a unique fictional identity (Lisa, Anna, Paul, and Peter) that they portrayed consistently in all of the recorded scenarios. To create different relationship types between the actors, they were paired to construct a mixed sex couple (Paul and Lisa), female friends (Lisa and Anna), mixed sex colleagues (Anna and Peter), or a male boss/employee (Paul and Peter, see Fig 1). Each actor thus appeared in half of the recorded vignettes, although not always with the same person, and they did not appear with certain characters at all as defined by the four relationship types. In total, 960 video vignettes varying in duration between 3 and 20 seconds were recorded (48 scenes x 5 intentions x 4 relationships). In addition, an introductory video was recorded (duration = 53 seconds) that identified each character by face and name, providing explicit details of their social role and relationship with other characters, to show to participants who were recruited to judge the meaning of the stimuli in the context of these details.

Individual scenes always consisted of interactions between two people which ended in a literal (positive or negative), jocular, sarcastic or white lie response; in other words, the speaker’s intention was to: (1) tell the truth, (2) use jocularity to be humorous, (3) amplify a negative opinion of the truth by making a critical counterfactual comment, or (4) conceal the truth in a white lie. Written scripts were first developed by the principal author (KR) for each of the 48 scenes (example scripts can be found in Table 1; example videos are available in the supplementary section).Topics of conversation included expression of opinions (about objects, places, people) and discussion of common situations (e.g., asking for help). To provide contextual cues that would allow participants to understand the true intentions of the speaker who uttered the response (especially for nonliteral meanings), approximately half of the scenes began with one of the characters talking to an unseen third person on the phone where they revealed their true state of mind (e.g., “I really didn’t like that restaurant he recommended”) before interacting with the character who posed the question. To ensure that scripts could be acted out in a manner that seemed natural in conversation, and that they reflected common language usage, they were thoroughly reviewed by a small pilot group (six native speakers of Canadian English, departmental colleagues). This process yielded minor modifications in wording and in the structure of the interactions prior to giving actors the scripts during the recording sessions.

### Recording procedures

All videos were recorded in a large sound-attenuated laboratory (McGill Language Acquisition Lab) using a Sony HDR-PJ580V HD camera. Two different physical scenes were created in the laboratory space, one depicting an office environment (with two chairs, a desk, desktop computer, and telephone) and one depicting a living room (with small couch and table). Scenes involving the couple and the female friends always took place in the living room set, whereas the colleague and the boss/employee scenes were situated in the office set. In addition, three distinct physical configurations (camera angles) were used depending on the physical scene (office or living room) and whether context was available; these differences affected the visibility of the two actors across scenes, although these factors were held constant for the different intentions that were communicated in each setup. The three configurations were: (1) a frontal view of only the actor delivering the final critical utterance was visible on the screen (n = 21; two items with context; office and living room scenes); (2) a frontal view of both actors who were side-by-side (n = 12; all items with context; office and living room scenes); (3) a frontal view of the actor delivering the final critical utterance and a back (over-the-shoulder) view of the second actor (n = 15; thirteen items with context; office scenes only). At the onset of recording, the four actors were given the written scripts and instructed to study them in advance; they were then encouraged to act out the different intentions as naturally as possible. Actors were not provided specific cues or instructions on *how* to enact the different intentions (e.g., to use specific facial expressions) and they were allowed to make minor changes to the wording in the script if it felt more natural to them, and to repeat scenes, although they were not permitted to change the wording of the final critical response.

Each recording session lasted around two hours, with 3–4 sessions required to complete recordings for each relationship type (recordings were blocked by relationship type as a practical measure to schedule specific actors for the recording sessions). Under the direction of the first author (KR), all scenes were filmed by a technical assistant with experience as an amateur filmmaker, who edited the digital recordings for the perception study. After each recording session, videos were backed up on a transportable hard disk and then cut and saved as.mpg files. Both during and after the recording sessions, videos were carefully monitored to ensure: (a) the target intention was delivered in a natural way; (b) the complete scene was recorded from beginning to end without any disruptions or technical errors; and (c) the wording only included minor modifications in the verbal context and question part of the scene, and no modifications in the critical utterance. If one or more of these criteria were not fulfilled, the scene was repeated either during the recording session or in a subsequent session. At a final stage, a brief fade-in from black sequence was added to the beginning of each edited vignette to avoid an abrupt onset of the scene.

### Perceptual validation study

#### Ethics Statement

This study was ethically approved by the McGill Faculty of Medicine Institutional Review Board in accordance with principles expressed in the Declaration of Helsinki. Informed written consent was obtained for each participant prior to their involvement in the research.

#### Participants

Thirty-eight young adults (18 male, 20 female; mean age = 23.21 years, SD = 3.88) were recruited through the McGill classified ads to judge the video vignettes. All were native speakers of North-American English and each received CAD $50 compensation at the end of the experiment. After testing, seven participants (6 male, 1 female) were subsequently excluded because they did not follow instructions on how to respond (n = 3), or it was discovered after testing that they were not a native English speaker (n = 1) or performed the task under the influence of narcotics (n = 3). Results are therefore reported for a final sample of 31 participants (12 male, 19 female).

Previous studies suggest that there is a positive relationship between the ability to comprehend sarcasm and lies and, for example, the ability to adopt emotional perspectives and show empathic concern in real life, as measures by tools such as the Interpersonal Reactivity Index (IRI [78,79]). As such, participants filled out two questionnaires that provide information on their social communication skills before rating the videos: the Social Norm Questionnaire [80]; and the IRI [78,79]. The SNQ consists of 20 yes-no questions on social behavior; participants indicate whether a behavior would be appropriate in the presence of an acquaintance (not a close friend or family member) based on current social norms. The IRI [78,79] is a measure of dispositional empathy that conceptualizes empathy as a set of separate but related constructs, indexed by four seven-item scales (perspective taking, empathic concern, personal distress, fantasy scales). For the SNQ, participants had a mean score of 20.0 out of 22.0 (SD = 1.83; range 15–21), indicating high awareness and knowledge of social rules in most participants. For the IRI, overall scores ranged from 47.0 to 86.0, with a mean of 66.65 (SD = 8.43). High scores (maximum: 112.0) indicate high empathy, whereas low score reflect low empathy. When compared to other studies testing larger populations [81], average empathy scores in the current sample are slightly higher [M = 58.0; see (81) for details], and none of the empathy subscales showed problematic skew or kurtosis statistics, demonstrating sufficiently normal distributions.

#### Procedure

A total of 926 videos were entered into a perceptual experiment controlled by Windows Movie Maker (920 critical videos and 6 training videos). Stimuli were arranged in 20 blocks presented in a pseudo-randomized order with the condition that no three stimuli of the same intention and relationship type could appear in sequence. Each block consisted of 46 items, one taken from each of 46 different scenarios (the remaining two scenarios were used for the training block, see below). Due to the large number of stimuli, the experiment was completed during two testing sessions lasting 2.5 hours each, held on consecutive days, with each participant judging all of the items.

Participants were tested in a group of 38 individuals, seated in a classroom, with all videos being projected onto a large screen at the front of the room. Audio was presented free-field at a comfortable listening level through ceiling-mounted speakers. After completing the questionnaires (during the first session), participants were given a set of answer sheets with numbered fields that corresponded to the number of each video that was presented in sequence. They were told that the study focuses on how people communicate with each other and instructed to attend carefully to each social exchange and then make a two-part judgment after watching each video clip. First, participants answered a simple yes/no question to ensure that they paid attention to the content of the scene (e.g., “*Did Lisa like Anna‘s cookies*?”; Response alternatives = *Yes*, *No*). A second question asked them to identify the specific intention of the actor who uttered the final comment in the scene (e.g., “What did Anna intend by the last sentence?” Response alternatives = *Be sincere*, *Be sarcastic*, *Joke around*, *Tell a white lie*). Participants were instructed to select only one response option for each question by ticking the appropriate box. During the response phase, videos were paused until all participants were ready to proceed to the next item (approx. 6 seconds). The experiment began with a practice block of six items to get accustomed to the procedure prior to starting the actual experiment. At the end of the second session, participants filled out a post-experiment questionnaire to gather information about: (1) their knowledge of different audio-visual cues referring to speaker intentions and if they paid attention to them during the experiment; (2) their own use of sarcasm, jocularity and white lies in daily life and what they think their function is; (3) whether they perceived differences between scenarios based on the actors’ gender or the type of relationship; and (4) if they found anything unusual about the scenes, and if so, to specify this.

## Results

Responses to the initial “content” question, which probed whether the 31 participants were paying attention to what happened in the scene, were very accurate overall (M = 90% correct, SD = 4.0). Nonetheless, items that yielded an incorrect response on the content question were excluded from analyses of how speaker intentions were understood to eliminate any responses that could be the result of guessing or inattention.

### Recognition by intention type

Table 2 supplies the raw accuracy data (percent correct target identification) for each of the four intentions by relationship type, as well as corresponding unbiased recognition (Hu) scores that individually correct for how many stimulus categories and response possibilities are allowed in a forced-choice task [see 82].

**Table 2:** Proportion of correct responses (raw hit rates) and corresponding Hu scores (corrected for individual response bias) for stimuli encoding each intention, according to participant sex and the relationship type depicted in each video. Standard deviations are shown in parentheses.

Intention	Female Participants				Male Participants			
	Relationship				Relationship			
	Couple	Friends	Colleagues	Boss	Couple	Friends	Colleagues	Boss
***Raw scores***								
Literal Positive	0.90 (0.02)	0.93 (0.03)	0.95 (0.04)	0.91 (0.07)	0.82 (0.08)	0.86 (0.10)	0.89 (0.06)	0.85 (0.08)
Literal Negative	0.92 (0.06)	0.95 (0.05)	0.95 (0.04)	0.94 (0.06)	0.91 (0.03)	0.93 (0.05)	0.93 (0.05)	0.91 (0.07)
Literal Total	0.91 (0.04)	0.94 (0.03)	0.95 (0.03)	0.93 (0.06)	0.87 (0.06)	0.89 (0.08)	0.91 (0.05)	0.88 (0.07)
Sarcasm	0.82 (0.10)	0.86 (0.08)	0.81 (0.11)	0.79 (0.12)	0.70 (0.09)	0.71 (0.11)	0.70 (0.12)	0.66 (0.14)
Jocularity	0.89 (0.07)	0.89 (0.07)	0.82 (0.11)	0.87 (0.07)	0.74 (0.16)	0.74 (0.14)	0.75 (0.14)	0.78 (0.12)
White lie	0.82 (0.06)	0.86 (0.06)	0.84 (0.07)	0.85 (0.07)	0.74 (0.09)	0.76 (0.11)	0.75 (0.13)	0.77 (0.12)
***Hu Scores***								
Literal Total	0.91 (0.04)	0.93 (0.03)	0.92 (0.04)	0.91 (0.05)	0.87 (0.04)	0.88 (0.05)	0.88 (0.04)	0.87 (0.04)
Sarcasm	0.71 (0.09)	0.79 (0.1)	0.69 (0.13)	0.71 (0.14)	0.58 (0.08)	0.65 (0.16)	0.61 (0.16)	0.57 (0.16)
Jocularity	84.8 (12.2)	84.2 (13.2)	82.2 (13.8)	83.6 (14.0)	0.81 (0.09)	0.82 (0.13)	0.81 (0.10)	0.85 (0.08)
White lie	0.71 (0.10)	0.80 (0.07)	0.75 (0.10)	0.73 (0.10)	0.59 (0.10)	0.67 (0.11)	0.63 (0.11)	0.61 (0.11)

Inspection of trends in the raw data revealed that overall accuracy for the 31 participants was high (Mean = 85%, SD = 13), with notable differences in participants’ categorization of intentions depending on their sex; overall, women achieved higher accuracy scores than men (female mean: 82%, SD: 11; male mean: 73%, SD: 15). As shown at the top of Table 2, participants tended to detect literal meanings better than nonliteral meanings, while differences between literal positive and literal negative tokens appeared to be minimal. Sarcastic scenes were associated with the lowest accuracy overall when compared to other intentions. Moreover, the data imply that the type of relationship had the greatest influence on how well sarcastic intentions were recognized, with higher accuracy when the social interaction involved friends.

**Fig 2:**
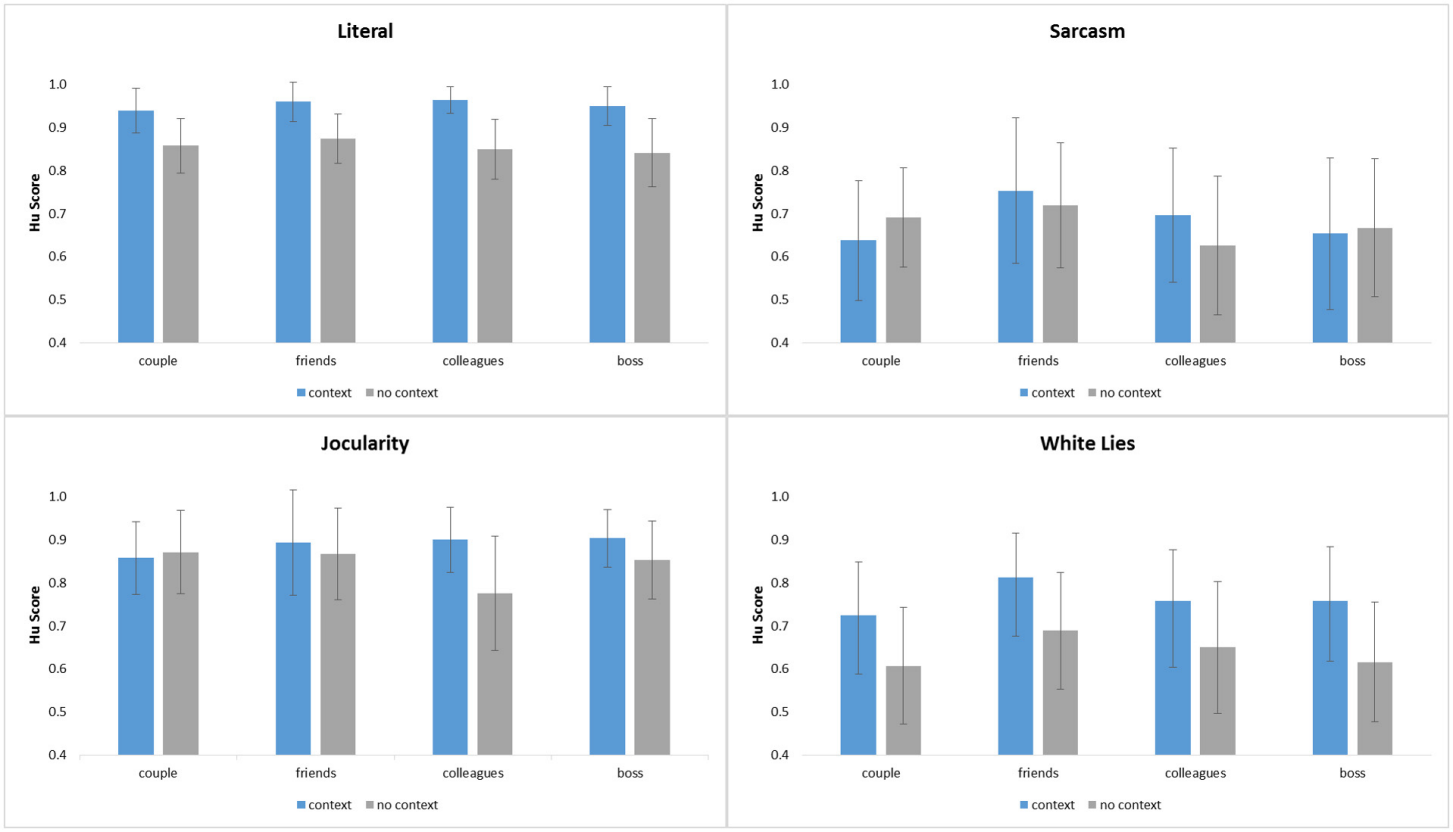
Accuracy in Hu scores. Hu scores for scenes with and without verbal context, displayed by intention type and relationship.

Statistical analyses were performed solely on the unbiased accuracy scores (Hu scores) summarized in Table 2 as well as Fig 2; note that calculation of Hu scores involved collapsing across literal positive and literal negative items, since the validation experiment required participants to decide between *four* different intentions (literal, jocularity, sarcasm, white lies), while correcting for the imbalance of tokens representing each intended response category in the perceptual experiment (i.e., literal = 368 items, jocularity, sarcasm, white lies = 184 items per category). The unbiased recognition scores were submitted to a 4 x 4 x 2 ANOVA with repeated factors of INTENTION (literal, sarcasm, jocularity, white lie), RELATIONSHIP (couple, friends, colleagues, boss/employee) and CONTEXT (verbal context, no verbal context). The ANOVA revealed main effects of INTENTION (F(3,28) = 84.15,p < .001), RELATIONSHIP (F(3,28) = 16.90,p < .001), and CONTEXT (F(1,30) = 95.53,p < .001). Pairwise comparisons to explore the INTENTION main effect confirmed that participants were significantly better overall in recognizing literal (M = 0.90) versus nonliteral utterances (sarcasm: M = 0.68; white lies: M = 0.70); jocularity (M = 0.86) was also recognized significantly better than sarcasm and white lies. In addition, speaker intentions were recognized significantly better overall when social partners were friends (M = 0.82) compared to all other relationship types (which did not differ; couple: M = 0.77; colleagues: M = 0.77; boss: M = 0.78), and scenes *with* verbal context promoted more accurate recognition of intentions (M = 0.82) when compared to scenes without context (M = 0.75).

The ANOVA also produced interactions of INTENTION x CONTEXT (F(3,28) = 20.89,p < .001), INTENTION x RELATIONSHIP (F(9,22) = 6.45,p < .001), and a three-way effect of INTENTION, RELATIONSHIP and CONTEXT (F(9,22) = 5.16, p < .001). The interaction revealed that while the impact of relationship type on intention recognition showed a general advantage for friends, this differed according to what specific intentions were being communicated. Pairwise comparisons showed that three intention types were recognized best when communicated by friends: literal meanings were detected more accurately for friends than for the couple or boss/employee; sarcastic meanings were identified best for friends than all other relationship types; and white lies were recognized best between friends than between colleagues or the boss/employee. In contrast, the response pattern to jocular utterances revealed a significant advantage for *colleagues* when compared to boss/employee interactions (see Fig 2). In terms of the facilitative effects of verbal context, note that scenes *with* verbal context were recognized better than those without context across relationship types only when processing literal meanings and white lies; although always in the same direction (context > no context), these patterns were more selective for sarcasm (only couple and colleague interactions) and jocularity (only colleague, boss/employee interactions).

**Fig 3:**
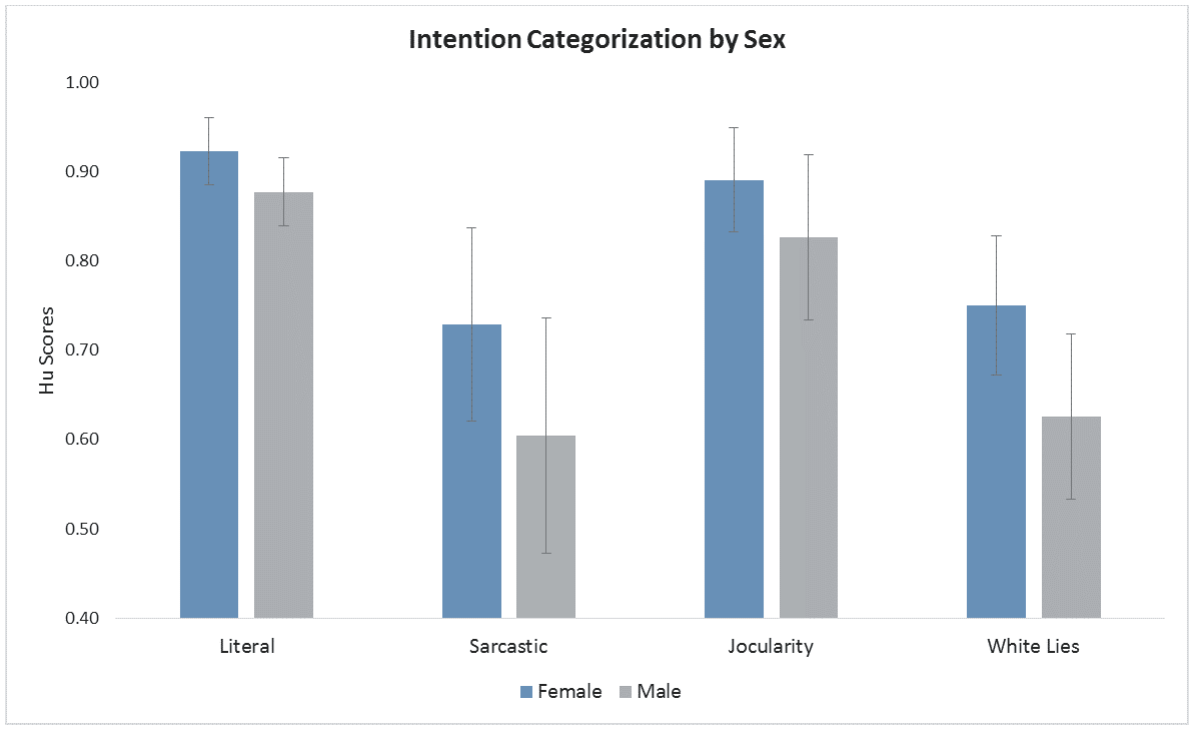
The effect of sex. Hu scores for female and male participants by intention and relationship.

As data in Table 2 implied that categorization of intentions differed by sex, an ANOVA with the factors INTENTION, RELATIONSHIP and participant SEX was subsequently performed. This analysis produced a significant main effect of SEX (F(1,29) = 11.16, p < .01), confirming superior recognition of speaker intentions overall by female when compared to male participants. However, the interaction of INTENTION x SEX (F(3,27) = 5.57, p < .01) revealed that the female advantage in intention recognition was only significant for sarcasm and white lies (see Fig 3).

### Relationship between intention recognition and social sensitivity/empathy

To examine whether individual differences influenced the ability to recognize speaker intentions in the validation task, we performed two-tailed Pearson correlations on the Hu-Scores between the four different intentions (literal, sarcasm, jocularity, white lies), the overall score all intentions combined, and the total scores of the Social Norm Questionnaire and Interpersonal Reactivity Index (including all subtests). No significant correlations were observed for these data.

### Qualitative data on intention recognition

After the validation was finished, subjects answered several questions about the videos using a post-experiment questionnaire, to gather subjective data on how they *believed* they judged speaker intentions during the experiment. A summary of responses to the question “Which cues did the protagonists use to signal their intentions (i.e. tone of voice, facial expressions etc.)?” can be found in Table 3.

**Table 3:** Responses on the post-experiment questionnaire to the question, “Which cues did the protagonists use to signal their intentions (i.e. tone of voice, facial expressions etc.)”. Comments are arranged by frequency of reported occurrence for each type of cue.

		Type of Cue Reported	
Intention	Vocal Cue	Facial Cue	Body Language
Sarcasm	**Some change in tone of voice (e.g., “negative”, “passive-aggressive”, “arrogant”)** (17)	1 **Comments on facial expression; e.g. rude, annoyed, exaggerated** (14)	**Hand gesture** (2)
	Loud/deep voice (5)	Eye rolling (8)	Shaking head (1)
	Exaggerated intonation (5)	Vocal cues (such as tone or word choice) did not match with facial cues (such as expression) (3)	
	Emphasis on particular words (4)	Scrunched, disgusted face (3)	
	Monotone, emotionless voice (2)	Glares/grimaces (3)	
	Slower speech rate (2)	Eye brows raised (2)	
	Slight upward inflection (1)	Maintained eye contact (1)	
		Wide eye (1)	
Jocularity	**Laughing/giggling** (20)	**Smiling** (5)	**Nudging** (2)
	Fake accent (4)	General facial expression (4)	Hand gestures (2)
	Tone of voice (8): using words like silly, dramatic (3)	Changing expression from serious to soft (2)	
	Exaggerated intonation (3)		
	Slowing words (1)		
	Pausing after statement (1)		
White Lies	**Hesitation, pauses, delay in response** (10)	**Avoiding eye contact, gaze away** (14)	**Stiff body** (1)
	Voice tone (9): used words such as artificial, upbeat (5)	Smiling (8)	Timid/sheepish (1)
	Monotone voice (2)	Fake smiles (5)	
	Increased pitch (2)	General facial expression (4)	
	Softer, slower voice (2)	Surprised face (1)	
	Emphasizing certain words (1)		

### Defining the RISC database: selection of a controlled subset for future studies

Based on perceptual data from the validation study, we identified a subset of tokens (30 unique scenes, 15 with context, controlled for actor visibility) that received high accuracy ratings that should be ideally used for testing or assessment purposes. Each of these scenes was associated with a minimum 75% mean accuracy level of the target intention across relationship types, yielding a database of 600 tokens in total (30 scenes x 5 intentions x 4 relationship types). Features of the refined RISC database are shown in Table 4 and these stimuli are freely available for download via the following webpage: https://mcgill.ca/pell_lab/neuropragmatics-and-emotion-lab-pell-lab.

**Table 4:** Average Hu accuracy scores (with standard deviation) for stimuli included in the refined RISC database, by intention and relationship type.

Intention	Number of Items	Relationship Type			
		Couple	Friends	Colleagues	Boss
Literal (120 Positive + 120 Negative)	240	0.91 (0.06)	0.92 (0.06)	0.92 (0.06)	0.90 (0.06)
Sarcasm	120	0.66 (0.14)	0.73 (0.17)	0.68 (0.17)	0.67 (0.18)
Jocularity	120	0.91 (0.09)	0.90 (0.12)	0.88 (0.10)	0.89 (0.08)
White lie	120	0.68 (0.14)	0.73 (0.13)	0.70 (0.13)	0.69 (0.14)

## Discussion

The ability to accurately process social information is crucial to predict other peoples’ behavior and to act accordingly; it also plays a central role in human development, enabling us to adjust to an increasingly complex social environment as we mature to adulthood [1,83,84]. According to socio-pragmatic views of communication, processing the literal meaning of an utterance is often insufficient to understand what is really *meant* by the utterance [17], as the interpretation of conversational remarks can depend highly on nonverbal cues[8,43,85] and key features of the social environment [5]. Thus, research that seeks to advance ideas about nonliteral language processing, such as jocularity, sarcasm and white lies, requires enriched materials that mark these intentions by incorporating important extra-linguistic cues such as speech prosody and body language as well as social context (e.g., knowledge of different relationship types). As an outcome of developing the RISC database, it is hoped that researchers can examine how speaker intentions are understood by different participant or clinical groups, and how different input channels are used when making social inferences. As well, these stimuli can facilitate theoretically motivated psychological and neuroscientific investigations of the processes underlying the perception of sincerity, jocularity, sarcasm, and white lies. In what follows, we discuss key features of our stimuli in light of our perceptual data and the broader literature.

### Recognizing literal versus nonliteral speaker meanings

The results of our validation study show that most participants had no trouble responding to the initial “content” question, which probed whether they were paying attention to what happened in the scene (M = 90%). In response to the categorization question, participants also did quite well in recognizing and categorizing the different speaker intentions (mean accuracy above 80%), although significant differences between literal and nonliteral items emerged. Overall, our data show that participants were more accurate to identify literal and jocular statements when compared to white lies and sarcastic utterances [76,86]. The higher accuracy scores for literal scenes might be due in part to their *unmarkedness*–being sincere can be seen as the default intention [80], fulfilling basic Gricean conversation maxims. In his model of conversation [87], communication is described as a contract between cooperating equals who set out to transmit information in the most honest, clear and efficient manner possible. Thus, detecting sarcasm and white lies requires sensitivity to a violation of the conversational “maxim of quality” [which assumes that all speekers are being truthful; 87]. Additionally, the better performance for literal utterances is in line with previous findings of an ostensible “truth bias” in communication [55,88–90]. This bias has shown to be influenced by the relationship between communication partners, with closer relationships leading to a stronger truth bias [91]. As DePaulo and Kashy [47] pointed out, close relationships usually incorporate ideals such as openness and authenticity, therefore leading to high expectations about sincerity and truthfulness. Similarly, McCornack and Parks [92] suggested that a truth bias is an integral part of maintaining intimacy in close relationships. The relationship types used in the RISC database tend to have a stronger predisposition towards closeness, with the boss/employee relationship as an exception due to the implied authority/power difference. It is therefore not surprising that recognition is improved for literal (sincere) interpretations of speaker intentions; moreover, our data imply that relationship status had an effect on identifying literal statements in the current validation study, leading to higher accuracy scores for closer relationships, especially friends (see Table 2 as well as Fig 2).

When comparing how nonliteral meanings are recognized, participants performed better at recognizing jocularity when compared with sarcasm and white lies. This result is not surprising, since white lie scenes are expected to be harder to identify [88,93]. In comparison to jocularity and sarcasm, individuals making white lies usually want to hide their true intentions and the cues that reveal this intention may therefore be very subtle [7]. However, participants were also less accurate at identifying sarcasm when compared to jocularity. This result is likely explained by the type of cues used by actors to signal these intentions, particularly for jocularity; in most of the jocularity scenes, actors spontaneously laughed, often at the end of the critical utterance, as an obvious ‘play cue’ to indicate that they were clearly “joking around” [see 94]. It has also been suggested that some individuals are more sensitive to ironic intent than others, perhaps by virtue of being frequently ironic themselves [95]; as a consequence, these individuals may detect jocularity in situations where other listeners might not. Although we found no obvious relationship between measures of inter-personal sensitivity and the ability to categorize speaker intentions, our study is based on an insufficient sample of participants to evaluate these effects thoroughly. Future studies that employ our stimuli should therefore look deeper at how individual factors influence how speaker meanings are understood. Of particular interest, previous studies have shown that the perception of sarcasm depends on whether participants are asked to judge *speaker intent* (i.e., the underlying motivation of the speaker) versus *social impressions* of the statement on[the impression that a statement creates for the addressee, (33)]. Asking participants about feelings, emotions or impressions is likely to engage Theory of Mind processes (such as perspective taking and empathy) to a greater extent, and might therefore lead to a stronger influence of individual differences on the perception of speaker intentions.

### The role of verbal context

In order to comprehend nonliteral language, additional complexity seems to arise by the amount and kind of contextual information that is required to correctly recognize it. This relates particularly the consideration of pragmatic cues, such as prosody, facial expressions, and gestures. However, the verbal context of an utterance, such as previous conversations, is also an important source of information. In the current study, we decided to consider verbal context as a facilitator in the recognition of speaker intentions. As expected, the validation data shows that literal, jocular and especially white-lie statements are easier to identify when participants are given verbal context about the true feelings and opinions of the speaker. It seems that the verbal context creates expectancies about the following statement, which are used by participants to determine speaker beliefs, a finding that has been shown in earlier studies [96]. Research has also shown that irony, for example, is perceived as such partly as a result of incongruity between the verbal context and the actual statement [28,33]. Additionally, it has been suggested that the greater the incongruence between what the listener expects to hear and what they actually hear, the greater their likelihood to perceive a statement as sarcastic [95].

Interestingly, when looking at the effect of verbal context in the present study, it can be seen that sarcastic comments do not profit to the same degree from having more information about the speaker intention, i.e. having heard about their true opinions and feelings. This is surprising, as previous research has shown that verbal context plays an important role in sarcasm perception [13,61,97,98]. One theory of sarcasm perception, the *allusional pretense theory* [99], emphasizes the importance of verbal context and expectations in sarcasm perception. According to this theory, the necessary conditions to elicit sarcasm are allusions to failed expectations and pragmatic insincerity [99]. These are conditions that, according to the theory, must be present within the greater contextual information provided in order to achieve the desired understanding of the sarcastic utterance. While it is not immediately clear why participants here did not benefit from verbal context in the sarcastic condition, future studies employing these stimuli could shed light on this issue by comparing the *same* scenarios with and without verbal context, by cutting off the first part of the conversation in scenarios with context. It would also be interesting to manipulate expectancies for interpreting sarcastic statements by cross-splicing verbal contexts from different scenes. Another important aspect in this respect might be the amount of nonverbal cues available to participants, as displayed by both conversation partners. In scenes *with* verbal context, the questioner is often not visible, while in scenes without verbal context both actors are typically present. This might have consequences for the identification of sarcastic utterances, since participants seem to rely strongly on nonverbal cues for interpreting sarcasm (review Table 3). It would appear that sarcastic scenes may be richer in terms of visual and auditory nonverbal information, and that these interpretations are guided to a relatively larger extent by *nonverbal* as opposed to verbal context.

### The role of social relationships and sex

A key factor that we manipulated in RISC was the type of relationship that exists between communication partners. According to the literature, the status of a relationship has a major influence on how we perceive literal and nonliteral utterances [34]. Some forms of nonliteral speech are affiliative, whereas others are sources of estrangement between individuals [16,100]. The psycholinguistic literature has traditionally studied irony as cases where speakers utter sarcastic comments with negative, critical intent, but many instances of ironic language actual enable speakers to bond together through their disparagement of another person [101]. Here we manipulated the bond between communication partners, ranging from more distant (boss/employee) to closer relationships (couple). The results of the validation study revealed a significant effect of relationship type on the decoding of speaker intentions, although these effects were smaller than anticipated and mostly limited to the *friend* relationship where accuracy tended to be higher. This effect should be viewed with some caution, as this could be partly due to the acting ability of the two friends in our database who might have been better at communicating literal statements, sarcasm, and white lies. Nonetheless, this result fits with the literature suggesting that a solidary relationship between speaker and addressee facilitates the meta-representational inferences needed to understand certain forms of nonliteral speech [29]. The post-questionnaire data also supply insights into the role of relationship type; anecdotally, around one third of participants reported that they were influenced by relationship type when interpreting the scenes. For example, one participant commented that, “*The couple was quicker to instigate sarcasm while the co-workers were more delicate in their social interactions*”, and another said, “*When Paul was telling a white lie to Lisa*, *he was nicer/less obvious about it than with the others*”. These comments appear to further validate the idea that relationship type has an effect on how speaker intentions are perceived and interpreted.

An important factor for detecting effects of relationship type seems to be the task [29]. In the current study participants had to *categorize* the videos into different speaker intentions, and it seems that the pure *decoding* of verbal and non-verbal cues is not influenced extensively by social context (e.g. business versus romantic relationship). However, considering the literature on nonliteral speech acts, it seems that evaluation of the *social appropriateness* of the videos would be influenced to a much larger degree by relationship type. For example, Jorgensen [34] found that appropriateness ratings for the use of sarcasm differ significantly depending on the degree of social distance or intimacy in the relationship. Pexman & Zvaigzne [29] found that statements made to solidary addressees were perceived to be funnier, more teasing, and less status changing than statements made to non-solidary addressees. Another possible reason as to why we did not find larger differences for the different relationships is that, even though subjects were informed about the intended nature of the relationship, they often reported that “all actors (characters) seem like they are friends”. The fact that the boss/employee and colleagues engaged in frequent sarcastic/jocular interactions could have produced the impression that they were friends, rather than having a (distant) professional relationship, an unexpected outcome of participants becoming familiar with the videos in our inventory. Alternatively, this impression could represent a limitation of our dataset in that relationships constructed to be distant may not have been consistently perceived as such. Future studies should bear these issues in mind as they explore the impact of relationship type on inter-personal communication in the context of our stimuli.

The validation study also revealed differences in accuracy when comparing male and female participants, both in response accuracy as well as in the post-questionnaire. In response the role of *sex* in portraying the intentions, about a third of the participants reported differences, such as “Men were more subtle, women used more gestures” or “The women were generally more emotive. Sex differences in the perception of non-verbal cues are frequently reported in the literature, with superior performance for females over males [102–104]. According to Hall and Mast [105], women are more interpersonally sensitive than men, both as a general trait and as a more specific skill in terms of judging the meanings of nonverbal cues [106,107]; this could have facilitated the ability of female participants to detect speaker intentions for our stimuli. Interestingly, other research suggests that men are more likely to perceive sarcasm as an affirmative experience, whereas women tend to perceive it as offensive and bothersome [34,108]. Indeed, both male and female participants agree that sarcasm is the purview of males [72,109], suggesting that there are socially shared appraisals and stereotypes of what is and is not appropriate in conversation [105]. Furthermore, males are more likely to tease and insult [108,110], whereas women are more likely to avoid such face-threatening situations [111]. In terms of lying behaviour, it seems that men tell more self-oriented lies than women [45] and perhaps lie in different ways for different reasons [112]. Although our current analyses may not have been sensitive to many of these sex-related patterns that shape inter-personal interactions, these issues may well inform future studies that employ these materials.

### Effects of different nonverbal cues

It is clear that recognition of many speaker intentions depends strongly on accurate detection and integration of relatively subtle cues that occur in both the auditory and visual modalities, as the social and verbal context unfolds. As the usage of nonverbal cues was not scripted, coached or controlled in any way within the RISC database, it was possible to observe a number of spontaneous indicators corresponding to different intentions that may be of interest to future researchers. Interestingly, the results of a post-study questionnaire administered to our participants indicated that they subjectively felt that they relied on a number of specific facial, vocal, and bodily cues in order to arrive at an interpretation of speaker intention, many of which overlap with those previously mentioned in the literature ([8,85] see also review Table 3).

Of particular note, participants were aware that jocular utterances are usually accompanied by laughter, a common communicative strategy to assure that the negative content of the message is not taken literally [9]. Anecdotally, we found that jocularity was the hardest intention for actors to perform during the recording sessions, perhaps because jocular statements do not always fit coherently with the discourse context and are therefore sometimes unexpected or paradoxical [25,28]. Jocular utterances also seem to involve highly exaggerated intonation, in line with previous data on the acoustic correlates of irony [113,114]. In addition to these markers, some actors spontaneously adopted fake (i.e., non-native) accents, such as British English, to express jocularity, a potentially important cue that is rarely mentioned in the irony literature. Palmer [115] argued that paralinguistic features of humor include, "a light-hearted tone of voice (perhaps accompanied by a smile); a tone which is obviously inappropriate to what is being said or the circumstances…a fake accent…(and) the commonest of all…laughter". Although we did not undertake acoustic analysis of our stimuli in this study, it would appear that fake accents should be included in the repertoire of vocal cues that speakers can use to mark a teasing or mocking attitude [116], pending further research on this topic. Similarly, participants reported that they used auditory cues to recognize sarcastic intention (e.g., “a slight upward inflection”, “a passive-aggressive, arrogant tone“), in line with studies reporting a variety of acoustic modifications in the context of sarcastic speech [43,44,117,118]. As argued by Bryant [9], it is likely that vocal cues play an important role in the communication of nonliteral meanings, including various sub-types of irony (sarcasm, jocularity); however, there may not be specific acoustic targets that represent these meanings, but rather a set of contrastive cues that direct listeners to seek an alternative interpretation of the spoken utterance [8,44,118–120].

Another remarkable observation includes the apparent use of facial expressions, prosodic cues, and body language in white lie interactions, such as hesitations, stuttering, gaze avoidance, fake smiles and hanging shoulders (see Table 3). When people tell lies, they try to conceal their true intentions, resulting in rather subtle nonverbal cues that signal the lie [102]. This means that participants often perform only slightly better than chance when making judgments about truth or lies [88], although here, participants recognized lie interactions at a much higher accuracy level, possibly due to the available of multimodal cues to infer these meanings. In light of these details, it seems likely that the combination of visual and auditory cues in our videos aided participants in differentiating white lies from sincere and sarcastic interactions, as would the availability of discourse context which is often not present in many studies of lie detection. These ideas reflect further avenues for employing our stimuli in a more focused and systematic manner.

### Limitations, applications, and conclusions

The RISC database faces several limitations. Notably, one of the outcomes of controlling the lexical content across intentions within each scenario is that not all *scenarios* fit with all *intentions* in terms of perceived social norms of behaviour, as pointed out by several participants in the post-questionnaire (especially for certain nonliteral utterances). Some participants commented that sarcasm wasn't appropriate, or a white lie wouldn’t have been necessary, in certain scenarios, rendering the final remark highly unexpected. Along these lines, Kreuz & Glucksberg [28] emphasized that positive statements of irony (e.g. “You’re a fine friend”) can be easily used in most situations, whereas corresponding negative statements (e.g. “You’re a terrible friend”) are much more restricted and unexpected. Thus, it should be acknowledged that expectancies, in terms of the communicator, relationship, and social context, are likely to influence perception and interpretation of intentions for certain items in our database, a variable that should be subjected to further scrutiny. Another limitation is that some scenarios were set up in the way that the visual reaction of the questioner is not visible as the interaction unfolds (these scenes were shot with the questioner’s back to the camera or outside of the scene). For example, when Anna asks Peter if he noticed that she has been working out, and he answers “No, not really”, she expresses her frustration with his comment by reacting with a short disgruntled vocalization that is audible, although her face is not visible. While the reaction to critical utterances was not scripted or predetermined in any form, their visibility (or lack thereof) is likely to furnish additional cues to identify intentions displayed by the responder, although this was not tightly controlled across our items.

Another possible concern is that our main experiment was carried out in one large group of 38 participants; given the social nature of the study and materials, it cannot be discounted that there was an impact of participating in a large group, rather than in individual testing sessions, on our measures. For example, in a group situation, people are more likely to give an answer that they think is socially expected, rather than one that reflects their genuine opinion. However, since our participants completed answer sheets independently, without any contact with those around them, it seems unlikely that responses gathered for our stimuli were strongly influenced by social expectancies associated with the current testing environment, although this factor should be monitored further.

In terms of potential applications, we note increasing interest in past years in how speaker intentions are understood by individuals with psychiatric disorders (autism, schizophrenia), neurodegenerative disease (Alzheimer’s or Parkinson’s disease, fronto-temporal dementia), and traumatic brain injury [7,77,121–124]. Evaluating social cognition in these groups will require ecologically valid stimuli that resemble everyday events, as captured by RISC, where an understanding of sarcasm, jocularity, and white lies can be assessed in relation to deficits in other cognitive areas such as information processing speed, working memory, learning, and executive reasoning. Moreover, for researchers interested in how processing speaker intentions relate to underlying brain responses using electrophysiology (e.g., EEG/ERPs) or neuroimaging, the current database offers (a) a sufficient amount of trials needed for experiments using these approaches, and (b) the possibility to directly compare the same content with varying auditory and visual cues to literal and nonliteral conversations.

## Supporting Information

S1 File Example videos from the subset (40 videos, scenes 19 and 33) as well as the introduction video. (ZIP)

S1 Table Detailed data for each video in the subset. (XLSX)
